# The efficacy of *EGFR* gene mutation testing in various samples from non-small cell lung cancer patients: a multicenter retrospective study

**DOI:** 10.1007/s00432-014-1789-x

**Published:** 2014-08-03

**Authors:** Paweł Krawczyk, Rodryg Ramlau, Joanna Chorostowska-Wynimko, Tomasz Powrózek, Marzena Anna Lewandowska, Janusz Limon, Bartosz Wasąg, Juliusz Pankowski, Jerzy Kozielski, Ewa Kalinka-Warzocha, Aleksandra Szczęsna, Kamila Wojas-Krawczyk, Michał Skroński, Rafał Dziadziuszko, Paulina Jaguś, Ewelina Antoszewska, Justyna Szumiło, Bożena Jarosz, Aldona Woźniak, Wojciech Jóźwicki, Wojciech Dyszkiewicz, Monika Pasieka-Lis, Dariusz M. Kowalski, Maciej Krzakowski, Jacek Jassem, Janusz Milanowski

**Affiliations:** 1 Department of Pneumonology, Oncology and Allergology, Medical University of Lublin, Jaczewskiego 8 St., 20-954 Lublin, Poland; 2 E.J. Zeyland Wielkopolska Center of Pulmonology and Thoracic Surgery, Poznan, Poland; 3 Cardiothoracic Surgery Department, Poznan University of Medical Science, Poznan, Poland; 4 Department of Genetics and Clinical Immunology, National Institute of Tuberculosis and Lung Diseases, Warsaw, Poland; 5 Molecular Oncology and Genetics Units, Department of Tumor Pathology and Pathomorphology, Franciszek Lukaszczyk Oncology Center, Bydgoszcz, Poland; 6 Department of Biology and Genetics, Medical University of Gdansk, Gdansk, Poland; 7 Department of Pathology, Specialist Pulmonary Hospital of Sokołowski, Zakopane, Poland; 8 Department of Lung Diseases and Tuberculosis, Medical University of Silesia, Zabrze, Poland; 9 Regional Centre of Oncology, M. Kopernik Hospital, Lodz, Poland; 10 Mazovian Centre for Treatment of Lung Diseases and Tuberculosis, Otwock, Poland; 11 Department of Oncology and Radiotherapy, Medical University of Gdansk, Gdansk, Poland; 12 Department of Clinical Pathomorphology, Medical University of Lublin, Lublin, Poland; 13 Department of Neurosurgery and Pediatric Neurosurgery, Medical University of Lublin, Lublin, Poland; 14 Department of Clinical Patomorphology, Poznan University of Medical Science, Poznan, Poland; 15 Department of Thoracic Surgery and Tumors, The Ludwik Rydygier Collegium Medicum, Nicolaus Copernicus University, Bydgoszcz, Poland; 16 Department of Tumor Pathology and Pathomorphology, The Ludwik Rydygier Collegium Medicum, Nicolaus Copernicus University, Bydgoszcz, Poland; 17 Department of Lung Cancer and Chest Tumor, Maria Skłodowska-Curie - Memorial Cancer Center and Institute of Oncology, Warsaw, Poland; 18 Institute of Rural Health, Lublin, Poland; 19 Department of Tumor Pathology and Pathomorphology, The Franciszek Lukaszczyk Oncology Centre, Bydgoszcz, Poland

**Keywords:** Non-small cell lung cancer, *EGFR* gene, Mutation testing, TTF1, Tyrosine kinase inhibitors

## Abstract

**Introduction:**

Testing for the epidermal growth factor receptor (*EGFR*) gene mutations requires considerable multidisciplinary experience of clinicians (for appropriate patient selection), pathologists (for selection of appropriate cytological or histological material) and geneticists (for performing and reporting reliable molecular tests). We present our experience on the efficacy of routine *EGFR* testing in various types of tumor samples and the frequency of *EGFR* mutations in a large series of Polish non-small cell lung cancer (NSCLC) patients.

**Methods:**

Deletions in exon 19 and substitution L858R in exon 21 of *EGFR* gene were assessed using real-time PCR techniques in 1,138 small biopsies or cytological specimens and in 1,312 surgical samples.

**Results:**

Out of 2,450 diagnostic samples (containing >10 % of tumor cells), the occurrence of *EGFR* gene mutations was 9 %; more frequently in women (13.9 %) and adenocarcinoma patients (10 %), particularly with accompanying expression of TTF1 (13.0 %). The frequency of *EGFR* gene mutations was similar in cytological and histological specimens, and in primary and metastatic lesions, and did not depend on the percentage of tumor cells and quality of isolated DNA. Cytological or small biopsy, compared to surgical specimens showed lower percentage of tumor cells, with no impact on the quality of real-time PCR assay.

**Conclusion:**

Cytological and small biopsy samples with low (10–20 %) content of tumor cells and specimens from metastatic lesions are a sufficient source for *EGFR* mutation testing in NSCLC patients. The incidence of *EGFR* gene mutations in examined population was similar to those reported in other Caucasian populations.

## Introduction

Appropriate epidermal growth factor receptor (*EGFR*) gene mutation testing is an essential element in selecting non-small cell lung cancer (NSCLC) patients for therapy with tyrosine kinase inhibitors of EGFR (TKIs EGFR). Erlotinib and gefitinib (reversible TKIs EGFR) as well as afatinib (irreversible TKI EGFR) constitute effective first- and subsequent line management in advanced NSCLC patients with *EGFR* mutations. A series of phase III clinical trials showed higher response rates and longer progression-free survival in patients with *EGFR* mutations treated with first-line EGFR TKIs compared to platinum-based chemotherapy (Petrelli et al. 2012; Lee et al. 2013). Further, recent meta-analyses demonstrated the effectiveness of EGFR TKIs in second-line treatment in this subset of patients (Petrelli et al. 2012; Lee et al. 2013). Currently, there is no strong clinical and economic rationale for using EGFR TKIs in first- or second-line therapy in patients with wild-type *EGFR*. The activating *EGFR* mutations are the only independent predictors of TKIs EGFR activity. Although *EGFR* mutations occur more frequently in females, non-smokers and East-Asian patients, these factors should not be used for selection of patients for EGFR TKIs therapy (Petrelli et al. 2012; Lee et al. 2013; Travis et al. 2011). In many countries, *EGFR* mutational analysis is performed exclusively in patients with adenocarcinoma (AC) or adenosquamous carcinoma (ADSQ) in whom the *EGFR* gene mutations occur more frequently than in other NSCLC types. Higher incidence of *EGFR* mutations in lepidic non-mucinous, papillary and solid AC compared to invasive mucinous and acinar AC, as well as in tumors expressing thyroid transcription factor 1 (TTF1) is not well understood (Aisner and Marshall 2012; Eberhard et al. 2008).

*EGFR* mutations occur in approximately 10 % of Caucasian NSCLC patients. Deletions in exon 19 and substitution L858R in exon 21 account for approximately 90 % of *EGFR* mutations (Aisner and Marshall 2012; Eberhard et al. 2008; Rosell et al. 2009, 2012; Boch et al. 2013; Gahr et al. 2013). The rare *EGFR* mutations are scattered in exons 18–21 of tyrosine kinase domain of the *EGFR* gene. There are large discrepancies in the occurence of these mutations between particular clinical centers (Rosell et al. 2009, 2012; Boch et al. 2013; Gahr et al. 2013). This is mostly due to differences in selection of NSCLC patients for *EGFR* testing (patients with non-AC, patients with early stage NSCLC who underwent surgery, etc.). Another cause of these differences is most likely technical issues related to genotyping and the sensitivity of particular molecular methods may influence the effectiveness of *EGFR*. Direct sequencing technique requires more than 50 % of tumor cells in the specimen (e.g., surgical material), whereas real-time PCR and next-generation sequencing techniques (CE-IVD methods) allow *EGFR* detection in samples containing as few as 1 % tumor cells. Despite this, *EGFR* testing in low-cellularity specimens (small biopsies, cytological smears) is still being questioned (Aisner and Marshall 2012; Eberhard et al. 2008). Additionally, thermal and chemical treatment of cells or tissue specimens (formalin fixation and paraffin embedding) reduces the quantity and quality of DNA. In consequence in a proportion of cases *EGFR* mutations may be missed (Aisner and Marshall 2012; Eberhard et al. 2008).

The aim of this retrospective study was to assess the relationship between the type of examined material and the efficacy of *EGFR* testing in a large series of Polish NSCLC patients subjected to routine screening for TKIs EGFR therapy. We also estimated in this population the frequency of deletion in exon 19 and L858R substitution in exon 21 of the *EGFR* gene.

## Materials and methods

A total of 2,450 NSCLC patients (median age: 63 ± 9 years), including 1,503 males and 947 females from 10 Polish cancer centers were referred for routine *EGFR* testing during selection for EGFR TKIs therapy (Table 1). The type of NSCLC tumors was diagnosed by standard hematoxylin and eosin staining, and in the case of scarce material—by immunohistochemistry. Expression of TTF1 was assessed in 727 patients, p63 in 290, CK7 in 465 and CK5/6 in 192 patients. Surgical tissue samples from primary tumors, distant metastases and involved lymph nodes were collected from 1,138 patients, and small biopsy or cytological samples from 1,312 patients (Table 2).

**Table 1:** Patient characteristics

Histology	Gender		Age (median ± SD, years)	TTF1 (738)		CK7 (465)		CK5/6 (192)	
	Male 1,503 (61.3 %)	Female 947 (38.7 %)		Positive 585; (79.3 %)	Negative 153; (20.7 %)	Positive 444; (95.5 %)	Negative 21; (4.5 %)	Positive 35; (18.2 %)	Negative 157; (81.8 %)
Adenocarcinoma	1,188 (59.5)	807 (40.5)	63 ± 9	559 (81.5)	127 (18.5)	430 (96.2)	17 (3.8)	26 (14.7)	151 (85.3)
Large cell carcinoma	58 (67.4)	28 (32.6)	62 ± 8	17 (63)	10 (37)	8 (88.9)	1 (11.1)	2 (33.3)	4 (66.7)
NSCLC not otherwise specified	168 (66.7)	84 (33.3)	63 ± 9	0	0	0	0	0	0
Squamous cell carcinoma	77 (80.2)	19 (19.8)	64 ± 7	0	11	3 (50)	3 (50)	5	0
Adenosquamous carcinoma	12 (57.1)	9 (42.9)	68 ± 10	9 (64.3)	5 (35.7)	3	0	2 (50)	2 (50)

**Table 2:** The incidence of *EGFR* mutations according to gender, age, histology and type of examined material

Variable	Subset	*EGFR* wild type	*EGFR* mutation	*p*	Deletion in exon 19	L858R substitution in exon 21
All patients (*n*, %)	2,450 (100)	2,229 (91)	221 (9)		123 (5)	98 (4)
Gender (*n*, %)	Female (947; 38.7)	815 (86.1)	132 (13.9)	0.0001	73 (7.7)	59 (6.2)
	Male (1,503; 61.3)	1,414 (94.1)	89 (5.9)		50 (3.3)	39 (2.6)
Age (*n*, %)	≥63 years (1,286; 52.5)	1,159 (90.1)	127 (9.9)	0.12	68 (5.3)	59 (4.6)
	<63 years (1,164; 47.5)	1,070 (91.9)	94 (8.1)		55 (4.7)	39 (3.4)
Histology (*n*, %)	AC (1,995; 81.4)	1,796 (90)	199 (10.0)	0.001*	112 (5.6)	87 (4.4)
	LCC (86; 3.5)	81 (94.2)	5 (5.8)		3 (3.5)	2 (2.5)
	NSCLC NOS (252; 10.3)	244 (96.8)	8 (3.2)		4 (1.6)	4 (1.6)
	SCC (96; 3.9)	91 (94.8)	5 (5.2)		1 (1)	4 (4.2)
	ADSQ (21; 0.9)	17 (80.9)	4 (19.1)		3 (14.3)	1 (4.8)
Type of examined material (*n*, %)	Surgically resected material (FFPE blocks) (1,138; 46.4)	1,026 (90.2)	112 (9.8)	0.211	62 (5.4)	50 (4.4)
	Biopsy material (FFPE blocks, cellblocks, cytological smears) (1,312; 53.6)	1,203 (91.7)	109 (8.3)		61 (4.6)	48 (3.7)
	Surgically resected primary tumors (FFPE block; 705; 28.8)	630 (89.4)	75 (10.6)	0.513	46 (6.5)	29 (4.1)
	Surgically resected metastatic tumors (FFPE block; 256; 10.4)	231 (90.2)	25 (9.8)		9 (3.5)	16 (6.3)
	Surgically resected metastatic lymph nodes (FFPE block; 126; 5.1)	117 (92.9)	9 (7.1)		5 (4)	4 (3.1)
	Fresh frozen material from primary tumor surgery (51; 2.1)	48 (94.1)	3 (5.9)		2 (3.9)	1 (2)
	Endobronchial biopsy (FFPE block; 512; 21)	466 (91)	46 (9)		24 (4.7)	22 (4.3)
	Transthoracic biopsy (cellblock and cytological smears; 451; 18.4)	413 (91.6)	38 (8.4)		21 (4.7)	17 (3.7)
	EBUS-TBNA (cellblock and cytological smears; 349; 14.2)	324 (92.8)	25 (7.2)		16 (4.6)	9 (2.6)

*AC* adenocarcinoma, *LCC* large cell carcinoma, *NOS* not otherwise specified NSCLC, *SCC* squamous cell carcinoma, *ADSQ* adenosquamous carcinoma, *EBUS*-*TBNA* endobronchial ultrasonography-guided transbronchial needle aspiration biopsy, *FFPE* formalin-fixed, paraffin-embedded

* AC versus NSCLC NOS, *p* = 0.0004; AC versus LCC, *p* = 0.203; AC versus other NSCLC types combined, *p* = 0.0006

The molecular testing was performed in four genetic laboratories (Lublin, Warsaw, Gdansk and Bydgoszcz). DNA was isolated from formalin-fixed paraffin-embedded (FFPE) tissue sections or cellblock sections using QIAamp DNA FFPE Tissue Kit (Qiagen, Canada) and Cobas DNA Sample Preparation Kit (Roche, USA) in accordance with the manufacturer’s instruction. DNA from mechanically disrupted fresh frozen tissues and DNA from scrapped materials of tumor cells marked by pathologists in cytological smears were isolated using QIAamp DNA Mini Kit (Qiagen, Canada). The data on DNA concentration measured with spectrophotometry were available for 1,078 cases and were predetermined before *EGFR* testing. An A260/A280 ratio of 1.8–2 was considered as a pure and well quality of DNA and such DNA was qualified for *EGFR* testing. The late amplification plot of internal control (after 35 cycles) was defined as a “weak amplification” of internal control or positive sample in real-time PCR method.

EGFR Mutation Analysis Kit (Entrogen, USA) and real-time PCR technique were used for *EGFR* mutation analysis in 1,882 samples in Lublin and Bydgoszcz laboratories, whereas Cobas EGFR Mutation Test (Roche, USA) in 147 samples in Gdansk. Laboratory-defined assay and PNA–LNA PCR Clamp method in real-time PCR technique were applied to analyze *EGFR* gene mutation in 421 samples from Warsaw. All laboratories participated in External Quality Assessment (EQA) scheme for molecular genetic analysis of *EGFR* in non-small cell lung cancer organized by European Molecular Quality Network (EMQN). Despite of various methods using in individual laboratories, the percentage of patients with positive *EGFR* status was similar (insignificant differences). Hence, single technique (real-time PCR) was used for evaluation of *EGFR* mutations in all Centers.

Statistical analysis was performed using Statistica, version 10. Associations between *EGFR* mutations, clinical factors and the type of tumor material were examined using chi-square test. The Student’s *t* test was used for testing equality of population medians among groups. *p* values below 0.05 were considered significant.

The study was approved by the Ethical Committee of the coordinating center, the Medical University in Lublin (decision no. KE-0254/131/2011).

## Results

Activating mutations of *EGFR* gene were found in 9 % of NSCLC tumors and included deletion in exon 19 in 123 cases and L858R substitution in exon 21 in 98 cases (Table 2). The frequency of rare *EGFR* mutations was not shown due to differences in the type of examined rare mutations between centers involved in the study. *EGFR* mutations were observed significantly more frequently in women compared with men (*p* < 0.0001). There was a similar percentage of patients with *EGFR* mutations in younger (<63 years) compared to older patients and a median age of patients with and without *EGFR* mutations was exactly the same (63 years). *EGFR* mutations occurred more frequently in AC compared with other NSCLC types (*p* = 0.0008) and with not otherwise specified (NOS) NSCLC (*p* = 0.0007). However, *EGFR* mutations were found in all types of NSCLC and were surprisingly frequent in ADSQ (19.1 %). The feasibility of *EGFR* testing was similar in different types of material, and in samples from primary versus metastatic tumors. The mutations were slightly more frequent in surgical (9.8 %) compared to small biopsy and cytological specimens (8.3 %) (*p* = 0.211). *EGFR* mutations were significantly more frequent (*p* = 0.04) in surgically resected primary tumors (10.6 %) than in metastatic lymph nodes obtained by mediastinoscopy or endobronchial ultrasound transbronchial aspiration (7.7 %). The proportion between exon 19 deletion and exon 21 L858R substitution was independent of gender, age, NSCLC histology and the type of examined material (Table 2).

The expression of TTF1 antigen in non-squamous NSCLC was significantly more frequent (*p* = 0.043) in females (82.9 %) than in males (76.9 %) and did not depend on patients’ age. *EGFR* gene mutations were more frequent (*p* = 0.049) in TTF1-positive compared with TTF1-negative tumors (Table 3). The expression of other cancer tissue markers (CK7, CK5/6, p63) did not correlate with the occurrence of *EGFR* mutations.

**Table 3:** The incidence of *EGFR* gene mutations according to the expression of TTF1 in adenocarcinoma, large cell carcinoma and adenosquamous carcinoma

TTF1 expression	*EGFR* wild type (641; 88.2 %)	*EGFR* mutation (86; 11.8 %)	*p*	Deletion in exon 19 (52; 7.2 %)	L858R substitution in exon 21 (34; 4.6 %)
Positive (585; 80.6 %)	509 (87)	76 (13)	0.049	46 (7.9)	30 (5.1)
Negative (142; 19.4 %)	132 (93)	10 (7)		6 (4.2)	4 (2.8)

Most of molecular tests were performed on material containing ≥20 % of tumor cells (84.9 %) and with good amplification of positive internal control in real-time PCR assay (77.1 %; Table 4). However, *EGFR* mutations were detected with similar frequency (*p* = 0.495) in samples containing ≥20 and <20 % of cancer cells. *EGFR* mutations were found in 191 (9.2 %) cases out of 2,079 samples with ≥20 % tumor cells and in 30 (8.1 %) cases out of 371 samples of <20 % tumor cells. *EGFR* mutations were found in 9.5 and 7.5 % of cases with good and weak amplification of internal positive control in real-time PCR, respectively (*p* = 0.145). The total concentration of isolated DNA was 56.4 ± 226 ng/μl. DNA concentrations were similar (*p* = 0.54) in patients with wild-type and mutated *EGFR* gene (57.4 ± 228.18 and 51 ± 202.93 ng/μl, respectively).

**Table 4:** The cellularity of the specimen and DNA quantity and quality according to the type of examined material

Type of examined material	≥20 % of cancer cells (2,079; 84.9 %)	<20 % of cancer cells (371; 15.1 %)	*p*	Good DNA amplification (1,888; 77.1 %)	Weak DNA amplification (562; 22.9 %)	*p*	DNA concentration (median ± SD; ng/μl)	*p*
Surgically resected material (paraffin blocks)	995 (87.4)	143 (12.6)	0.001	872 (76.6)	266 (23.4)	0.633	88.6 ± 271.1	<0.0001
Biopsy material (paraffin blocks, cellblocks, cytological smears)	1,084 (82.6)	228 (17.4)		1,016 (77.4)	296 (22.6)		34.4 ± 103.2	
Surgically resected primary tumor (paraffin block)	598 (84.8)	107 (15.2)	<0.0001	500 (70.9)	205 (29.8)	<0.0001	104.5 ± 332.5	
Surgically resected metastatic tumor (paraffin block)	231 (90.2)	25 (9.8)		223 (87.1)	33 (12.9)		74.75 ± 130.8	
Surgically resected metastatic lymph nodes (paraffin block)	116 (92)	10 (8)		99 (78.6)	27 (21.4)		53.76 ± 134.0	
Fresh frozen material from primary tumors surgery	50 (98)	1 (2)		50 (98)	1 (2)		81.2 ± 242.3	
Endobronchial biopsy (paraffin block)	445 (86.9 %)	67 (13.1)		406 (79.3)	106 (20.7)		37.3 ± 106.2	
Transthoracic biopsy (cellblock and cytological smears)	362 (80.2)	89 (19.8)		357 (79.2)	94 (20.8)		26.7 ± 70.75	
EBUS-TBNA (cellblock and cytological smears)	277 (79.4)	72 (20.6)		253 (72.5)	96 (27.5)		36.6 ± 130.9	

*EBUS*-*TBNA* endobronchial ultrasonography-guided transbronchial needle aspiration biopsy

Scant cellularity was more frequent in biopsy specimens compared to surgically resected tissues (*p* = 0.001). Low content of tumor cells were most common in specimens from transthoracic biopsies and endobronchial ultrasonography-guided transbronchial needle aspiration biopsy (EBUS-TBNA). Sufficient PCR cycles number for amplification of internal positive control were similar in both surgical and biopsy specimens. However, a weak amplification of internal positive control was most common in surgically resected primary tumors and in EBUS-TBNA biopsies. Biopsy specimens, compared to surgically resected material contained significantly lower DNA concentration (*p* < 0.0001) (Table 4).

## Discussion

*EGFR* mutations occur more frequently in females, non-smokers and ACs (Petrelli et al. 2012; Lee et al. 2013; Rosell et al. 2009). The frequency of reported *EGFR* gene mutations in the European AC patients ranges from 8.7 % in Germany to 17.8 % in Spain (Rosell et al. 2009; Boch et al. 2013; Gahr et al. 2013). In these populations, the frequency of *EGFR* mutations in large cell carcinoma (LCC) patients was 11.5 and 3.6 %, respectively. The frequency of *EGFR* gene mutations in German squamous cell carcinoma (SCC) patients ranged from 0 to 3.5 % (Boch et al. 2013; Gahr et al. 2013). The frequency of L858R substitution was similar to the frequency of *EGFR* gene deletions. In the Spanish (AC and LCC) and German (AC only) populations, the *EGFR* gene mutations were more common in females (30 and 14 %, respectively) than in males (8.2 and 1.2 %, respectively) (Rosell et al. 2009; Boch et al. 2013). In the Spanish population, the percentage of *EGFR* mutations was independent of age: 13.9 and 18.9 % in patients below and above 56.7 years, respectively (Rosell et al. 2009). The differences between the frequencies of mutations in particular populations were mainly due to various criteria for selecting patients for *EGFR* testing, e.g., considering smoking history.

Our study includes the largest series of European NSCLC patients tested for *EGFR* mutations. The frequency of *EGFR* mutations (9 % in the entire population, 14 % in females and 10 % in AC) in patients considered to TKIs EGFR therapy was similar to that reported in the German population (Boch et al. 2013). Notably, we found a relatively high percentage of *EGFR* mutations in ADSQ (19.1 %) and in SCC (5.2 %) patients. It is likely that some SCC patients might have been misdiagnosed, as immunohistochemistry was not routinely performed. Moreover, the data on patient smoking status were not available in our analysis. Also, the weakness of our study results from the retrospective character of analysis.

There is an ongoing debate on whether the expression of immunomarkers, e.g., TTF1 on tumor cells could help to predict the *EGFR* mutations in AC patients. In Japanese AC patients, TTF-1 expression was found to be related to never smoking status and to the presence of *EGFR* mutations (Hiramatsu et al. 2010). The frequency of *EGFR* mutations in this study was 58 % in the entire group and 65 % in the TTF1-positive subset. In the German population, the occurrence of *EGFR* mutations was associated with TTF1 positivity and almost all *EGFR*-mutated NSCLC patients were TTF-1 positive (Tapia et al. 2009). Similar results were obtained by Leary et al. (Leary et al. 2012) who ascertained that all examined tumors with *EGFR* mutations were non-mucinous TTF1-positive. These three studies combined involved fewer than 500 patients. The results of our study including 727 patients confirmed these findings. However, in our material, *EGFR* mutations were also found in TTF1-negative tumors, suggesting that TTF1 negativity should not exclude patients from *EGFR* testing (Table 3).

There are only a few studies evaluating the presence of *EGFR* mutations in primary NSCLC and in corresponding metastases. In the study of Togashi et al. (2011), lung cancer metastases were diagnosed less frequently in wild-type *EGFR* compared to *EGFR*-mutated AC. However, in this study, the information about type of tumor samples (primary or metastatic) used for *EGFR* mutation analysis was not provided (Togashi et al. 2011). Sun et al. (2011), in a study including 80 patients showed full concordance of *EGFR* mutational status between 21 primary tumors and corresponding 26 lymph node metastases (Sun et al. 2011). We earlier found only 6.3 % (9/143) of *EGFR* gene mutations in the brain NSCLC metastases, with the full concordance with mutational status of primary tumor (Wojas-Krawczyk et al. 2013). In the current study, the frequency of *EGFR* mutations was similar in the primary and metastatic sites including lymph nodes (Table 2). This indicates that molecular testing may be reliably performed on both primary and metastatic tumors, depending on their availability.

It has been postulated that testing for *EGFR* mutations should use histological rather than cytological material (Billah et al. 2011; Smouse et al. 2009). Billah et al. (2011) concluded that 6.2 % (13/209) of cytological specimens (including 99 EBUS-TBNA specimens) were insufficient for molecular diagnosis, but they did not compare their results with corresponding surgical material. Moreover, the percentage of *EGFR* mutations in this series was relatively high (20 %). Smouse et al. (2009) reported the failure of molecular diagnosis in 3.5 % (8/227) and 8.3 % (1/12) of surgical and cytological specimens, respectively. However, the frequency of *EGFR* mutations was higher in cytological compared to surgical specimens (58 and 28 %, respectively). Esterbrook et al. (2013) showed that 11.2 % (4/36) of EBUS-TBNA samples were ineligible for *EGFR* mutation testing. Khode et al. (2013) found *EGFR* mutations in 28.6 % (16/56) of FFPE samples from resected tissues and biopsy specimens and in only 14.3 % (9/63) of cytological smears. This study also showed a high concordance of results obtained from cytological smears and FFPE samples achieved from the same anatomic sites. Additionally, the sensitivity of real-time PCR was lower than that of pyrosequencing platform (13 and 25 detected mutations, respectively). In a similar study, the overall *EGFR* mutation concordance between 60 histologic and corresponding cytological specimens was 91.7 % (Sun et al. 2013). More recently, Ma et al. (2012) tested *EGFR* mutations in 269 cytological specimens and in 1,141 surgically resected tissues from the Asian patients. *EGFR* mutations were found in 39 and 48 % of cytological and tissue specimens, respectively. An Italian study showed 9.7 % of *EGFR* mutations in fresh specimens obtained during CT-guided aspiration biopsy of peripheral AC (Stella et al. 2013). In another Italian study, including 92 cytological specimens, mutations were found in 24 % of the cases, and 12 % of samples were not assessable (Allegrini et al. 2012). All these studies were relatively small and used various laboratory techniques (e.g., macro- or microdissection) of DNA preparation and molecular testing. Our preliminary study (Krawczyk et al. 2012) (460 NSCLC patients) showed insignificantly higher in frequency of *EGFR* gene mutations in tissue (12.4 %) compared to cytological specimens (8.8 %). In another large study (755 NSCLC patients, *EGFR* testing was less successful in samples with low (<2 ng/μl) compared to those with high DNA concentration (70 vs. 96 %, respectively), with the corresponding *EGFR* positivity of 13 and 9.2 %, respectively (Leary et al. 2012).

It is generally believed that the quantity and quality of the material are crucial to perform a reliable *EGFR* mutation analysis. However, in the current study, including large series of routinely assayed NSCLC patients, *EGFR* mutations were detected with similar frequency in surgical and biopsy specimens, as well as in primary and metastatic sites. Further, the low percentage of tumor cells did not preclude effective *EGFR* analysis using real-time PCR techniques. Despite significant differences in tumor cellularity, the quality (amplification of internal positive control in real-time PCR) and the quantity of DNA obtained from different types of materials was similar, and so was the DNA concentration in samples with and without *EGFR* mutations. Our results confirm therefore that samples from small biopsies or cytology allow for reliable *EGFR* mutation testing.
